# Improving randomness characterization through Bayesian model selection

**DOI:** 10.1038/s41598-017-03185-y

**Published:** 2017-06-08

**Authors:** Rafael Díaz Hernández Rojas, Aldo Solís, Alí M. Angulo Martínez, Alfred B. U’Ren, Jorge G. Hirsch, Matteo Marsili, Isaac Pérez Castillo

**Affiliations:** 1 Instituto de Física, Universidad Nacional Autónoma de México, Apdo. Postal 20-364, Cd. Mx., C.P. 04510 Mexico; 2 Instituto de Ciencias Nucleares, Universidad Nacional Autónoma de México, Apdo. Postal 70-543, Cd. Mx., C.P. 04510 Mexico; 3 The Abdus Salam International Centre for Theoretical Physics, Strada Costiera 11, 34151 Trieste, Italy; 4 London Mathematical Laboratory, 14 Buckingham Street, London, WC2N 6DF United Kingdom

## Abstract

Random number generation plays an essential role in technology with important applications in areas ranging from cryptography to Monte Carlo methods, and other probabilistic algorithms. All such applications require high-quality sources of random numbers, yet effective methods for assessing whether a source produce truly random sequences are still missing. Current methods either do not rely on a formal description of randomness (NIST test suite) on the one hand, or are inapplicable in principle (the characterization derived from the Algorithmic Theory of Information), on the other, for they require testing all the possible computer programs that could produce the sequence to be analysed. Here we present a rigorous method that overcomes these problems based on Bayesian model selection. We derive analytic expressions for a model’s likelihood which is then used to compute its posterior distribution. Our method proves to be more rigorous than NIST’s suite and Borel-Normality criterion and its implementation is straightforward. We applied our method to an experimental device based on the process of spontaneous parametric downconversion to confirm it behaves as a genuine quantum random number generator. As our approach relies on Bayesian inference our scheme transcends individual sequence analysis, leading to a characterization of the source itself.

## Introduction

Random numbers have acquired an essential role in our daily lives because of our close relationship with communication devices and technology. There are also numerous scientific techniques and applications that rely fundamentally on our ability for generating such numbers and typically pseudo-random number generators (pRNGs) suffice for those purposes. A new alternative has been proposed by exploiting the inherently probabilistic nature of quantum mechanical systems. These Quantum Random Number Generators (QRNGs) are in principle superior to their classical counterparts and recent experiments have shown ref. 1 that they can reach the same quality as commercial pRNGs. However, the natural question of how to assess whether a sequence is truly random is not yet fully established. Pragmatically, the NIST test suite^2^ has become the standard method for analysing sequences coming from a RNG. The suite is based on testing certain features of random sequences that are hard to reproduce algorithmically, such as its power spectrum, longest string of consecutive 1’s, and so on. Even though it constitutes an easily applicable procedure, recent findings show that its reliance on *P*-values is a drawback^3, 4^, while its lack of formality is a major disadvantage. On the other hand, although no definition of randomness is deemed absolute, a rigorous characterization is presented by the Algorithmic Theory of Information (ATI) but it is unfortunately inapplicable in real cases^5^. An alternative which overcomes both formal and applicability issues is the Borel-normality criterion^6^ (BN). Intuitively, this approach works by successively compressing a given dataset, e.g. $\hat{s}=\{0101010010101010101011010\cdots \}$ of *M* bits, by taking strings of *β* consecutive bits and computing the frequency of occurrences ${\gamma }_{i}^{(\beta )}$ of each of those $i=0,1,\ldots ,{2}^{\beta }-1$ possible strings. For example, *β* = 1 corresponds to looking for the frequencies of the strings {0, 1} in the dataset $\hat{s}$, while *β* = 2 corresponds to analysing the frequencies of the strings {00, 01, 10, 11}, and so on. The whole sequence is said to be Borel-normal if the frequencies are bounded individually according to

1 $$|{\gamma }_{i}^{(\beta )}-\frac{1}{{2}^{\beta }}| < \sqrt{\frac{{\mathrm{log}}_{2}\,M}{M}},$$

and with *β* an integer ranging from 1 to *β*_max_ = log_2_ log_2_ *M*. It is important to mention that BN criterion is a (nearly) necessary condition for a sequence to be considered random^5^. Note that this test is restricted to a-single-sequence classification, so it cannot determine the random character of the generating *source*.

In the present work, we show that randomness characterization can also be addressed using a Bayesian inference approach for model selection^7^, borrowing the compression scheme of BN. For simplicity, for a fixed *β* we denote each string with its decimal base representation $j\in \{0,1,\ldots ,{2}^{\beta }-1\}\equiv {{\rm{\Xi }}}_{\beta }$. The first step consists in identifying the models which could have generated a compressed dataset $\hat{s}$. For instance if *β* = 1, we can describe it as *M* realizations of a Bernoulli process, leading to two possible models: with and without bias. Similarly, for *β* = 2, a model represents a way of constructing $\hat{s}$ with bias in some of the 2^2^ possible strings. A simple combinatorial counting reveals that all the possible bias assignations correspond to all partitions of the four strings of ${{\rm{\Xi }}}_{2}$.

Thus, in general, given the set ${{\rm{\Xi }}}_{\beta }$, let ${{\mathscr{P}}}_{{{\rm{\Xi }}}_{\beta }}$ denote the family of its $${B}_{{2}^{\beta }}={\sum }_{K=1}^{{2}^{\beta }}\,\{\begin{array}{c}{2}^{\beta }\\ K\end{array}\}$$ possible partitions^8^, with ${B}_{{2}^{\beta }}$ the Bell’s numbers and $$\{\begin{array}{c}{2}^{\beta }\\ K\end{array}\}$$ the Stirling numbers of the second kind, which counts the different ways of grouping 2^*β*^ elements into *K* sets. Formally, ${\alpha }_{\ell }^{(K)}=\{{\omega }_{\ell }^{\mathrm{(1)}},\ldots ,{\omega }_{\ell }^{(K)}\}\in {{\mathscr{P}}}_{{{\rm{\Xi }}}_{\beta }}$ would refer to the $\ell$-th partition into *K* subsets, but for notational simplicity we will omit henceforth the index $\ell$. To each partition *α* ^(*K*)^ there corresponds a unique model ${ {\mathcal M} }_{{\alpha }^{(K)}}$ which assigns a probability *p*_*j*_ to string $j\in {{\rm{\Xi }}}_{\beta }$ according to the following rule:

2 $${ {\mathcal M} }_{{\alpha }^{(K)}}=\{{p}_{j}=\frac{{\theta }_{r}}{|{\omega }^{(r)}|};\quad \forall r=1,\ldots ,K;\,\forall j\in {\omega }^{(r)}\}.$$

This means that all strings contained in a given subset *ω* ^(*r*)^ are deemed equiprobable within the specified model. Thus, keeping *β* fixed, the likelihood of observing the given dataset $\hat{s}$ in a model ${ {\mathcal M} }_{{\alpha }^{(K)}}$ is:

3 $$P(\hat{s}|{ {\mathcal M} }_{{\alpha }^{(K)}},{\{{\theta }_{r}\}}_{r=1}^{K})=\prod _{r=1}^{K}\,{(\frac{{\theta }_{r}}{|{\omega }^{(r)}|})}^{{k}_{{\omega }^{(r)}}},$$

where ${k}_{j}^{(\beta )}$ is the frequency of string $j\in {{\rm{\Xi }}}_{\beta }$ and we have defined ${k}_{{\omega }^{(r)}}={\sum }_{j\in {\omega }^{(r)}}\,{k}_{j}^{(\beta )}$ as the aggregate frequencies of the strings in the subset *ω* ^(*r*)^. (For further use, we also introduce the relative aggregate frequencies ${\gamma }_{{\omega }^{(r)}}=\tfrac{\beta }{M}{k}_{{\omega }^{(r)}}$). From this perspective, only the model that is symmetric under any reordering of the possible strings is identified with a complete random source, because any other model entails biases assignations according to the strings’ grouping represented by the corresponding partition. This symmetry only exists when the partition is the set ${{\rm{\Xi }}}_{\beta }$ itself, hence we denote ${ {\mathcal M} }_{{\alpha }^{\mathrm{(1})}}={ {\mathcal M} }_{{\rm{sym}}}$.

Consider now that when characterising randomness the only essential feature is whether bias for or against some strings is present, but the degree of bias is irrelevant. We can eliminate the dependence on the bias parameters by multiplying with a prior for ${\{{\theta }_{r}\}}_{r=1}^{K}$ and derive the so called *evidence* for a given model^9^. Following^10^, we use the Jeffreys prior for it yields a model’s probability distribution invariant under reparametrization and provides a measure of a model’s complexity, thus giving a mathematical representation of Occam’s Razor principle^10–12^. After integrating in the parameter space, we arrive at (see Supplementary Information (SI), Sec. 2)

4 $$P(\hat{s}|{{\mathscr{M}}}_{{\alpha }^{(K)}})=\frac{{\rm{\Gamma }}(\frac{K}{2})}{{{\rm{\Gamma }}}^{K}(\frac{1}{2})}\prod _{r=1}^{K}\,{(\frac{1}{|{\omega }^{(r)}|})}^{\frac{M}{\beta }{\gamma }_{{\omega }^{(r)}}}\frac{{\prod }_{r=1}^{K}{\rm{\Gamma }}(\frac{1}{2}+\frac{M}{\beta }{\gamma }_{{\omega }^{(r)}})}{{\rm{\Gamma }}(\frac{K}{2}+\frac{M}{\beta })}.$$

Eq. (4) is our main result, for it will let us perform the model selection straightforwardly. For ${ {\mathcal M} }_{{\rm{sym}}}$, its evidence is fairly intuitive:

5 $$P(\hat{s}|{ {\mathcal M} }_{{\rm{sym}}})\equiv P(\hat{s}|{ {\mathcal M} }_{{\alpha }^{\mathrm{(1)}}})={2}^{-M}.$$

Finally, we want to infer the model that best describes our source, *after* a dataset $\hat{s}$ is given. Using Bayes’ theorem the posterior distribution $P({ {\mathcal M} }_{{\alpha }^{(K)}}|\hat{s})$ reads:

6 $$P({ {\mathcal M} }_{{\alpha }^{(K)}}|\hat{s})=\frac{P(\hat{s}|{ {\mathcal M} }_{{\alpha }^{(K)}}){P}_{0}({ {\mathcal M} }_{{\alpha }^{(K)}})}{{\sum }_{\gamma }P(\hat{s}|{ {\mathcal M} }_{\gamma }){P}_{0}({ {\mathcal M} }_{\gamma })}.$$

Henceforth we will consider a uniform prior over models (which is justified in SI), so the model’s posterior is simply proportional to its evidence.

Suppose now we want to assess whether a source can be considered truly random. This is performed in two steps. As the first step, we need a model ranking procedure based on the posterior distribution. The second step consists in quantifying the goodness of our choice of model.

As a decision rule for the ranking process we use the Bayes Factor^13^ perspective,

7 $${{\rm{BF}}}_{\alpha ,\alpha ^{\prime} }=\frac{P({ {\mathcal M} }_{\alpha }|\hat{s})}{P({ {\mathcal M} }_{\alpha ^{\prime} }|\hat{s})}=\frac{P(\hat{s}|{ {\mathcal M} }_{\alpha })}{P(\hat{s}|{ {\mathcal M} }_{\alpha ^{\prime} })}.$$

Thus, we will choose ${ {\mathcal M} }_{\alpha }$ over ${ {\mathcal M} }_{\alpha ^{\prime} }$ whenever BF_*α*,*α*′_ > 1. It has been shown that BF_*α*,*α*′_ provides a measure of goodness of fit and ${\mathrm{lim}}_{M\to \infty }\,{{\rm{BF}}}_{\alpha ,\alpha ^{\prime} }=\infty$ if ${ {\mathcal M} }_{\alpha }$ is the true model^14^.

To implement the second step, which is nothing more than a hypothesis testing problem, we have two alternatives: either we check whether log_10_ BF_*α*,*α*′_ ≥ 2 which is considered decisive in favour of model ${ {\mathcal M} }_{\alpha }$ ^13^, or we compute the ratio between the posterior and the prior of a given model to assess how certain the posterior has become under the information provided by the dataset.

From a computational point of view notice that the evaluation of the posterior requires to being able to compute the normalization factor ${\sum }_{\gamma }\,P(\hat{s}|{ {\mathcal M} }_{\gamma }){P}_{0}({ {\mathcal M} }_{\gamma })$ that appears in (6). When the number of models is very large we can choose either to work with a subspace of models or use the logarithm of the Bayes Factor, as in this case the normalisation factor cancels out.

It is clear that a full test of randomness requires different values of *β* to be used for the same dataset, while the strings should be short enough so that the *M* bits allow for each of the possible models to be sampled at least once. Thus, heuristically, ${B}_{{2}^{{\beta }_{{\rm{\max }}}}}\sim M$ whence we can reproduce the BN limit^6^, *β*_max_ ~ log_2_ log_2_ (*M*), after using an asymptotic expansion for the Bell number.

**Figure 1:**
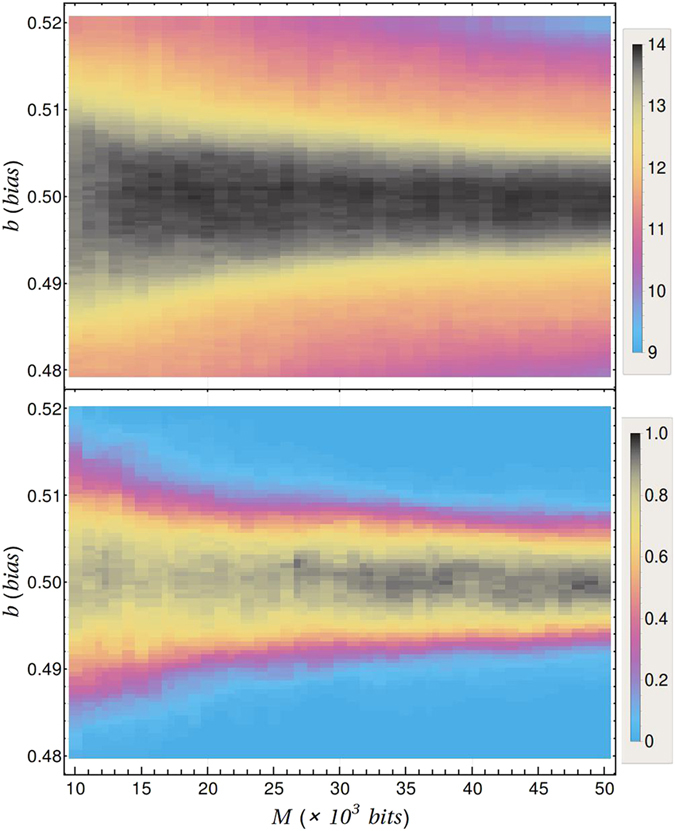
Phase diagram of Randomness Characterisation. Division of the parameter space into regions according to the likeliest model. The top figure corresponds to *β* = 1 in terms of the frequency *γ*_0_ of the string 0 and the sample size *M*. The green curves corresponds to Borel’s normality criterion, while the red curves are Borel-type bounds obtained by an approximation obtained from Eq. (4) (see Sec. 3 of SI). The bottom plot corresponds to *β* = 2 where each coloured area identifies the likeliest model in that region. Here we fixed the frequencies *γ*_1_ = 1/6 and *γ*_2_ = 1/4 and varied the frequency *γ*_0_ of the string 00 and the sample size *M*.

Note that by fixing *β* we have the set of parameters $({\{{\gamma }_{j}\}}_{j=0}^{{2}^{\beta }-1},M)$, whose space can be divided into regions identifying the likeliest model according to Eq. (4). As illustrative cases, in Fig. 1 we show a phase-type diagram for *β* = 1 and *β* = 2 (upper and lower panel, respectively), where the orange-filled area delimits the parameters values that renders ${ {\mathcal M} }_{{\rm{sym}}}$ the likeliest model. The top panel includes the bounds according to the BN criterion (green curves) given by Eq. (1), and shows that for any sequence length, M, our method allows for considerably smaller variations of *γ*_0_. This is a significant improvement, since only necessary criteria exist for testing randomness. The lower panel depicts the analogous regions when *β* = 2, for which there are fifteen models (see a list in the SI) and we have fixed two frequencies: *γ*_1_ = 1/6 and *γ*_2_ = 1/4. The complete models distribution can be deduced from the structure of this graph, by distinguishing, *a posteriori*, the equiprobable strings for which the corresponding model is the likeliest. Thus more information than complete randomness classification can be readily obtained from our method.

Also in Fig. 1, the red curves of the *β* = 1 case are bounds obtained by comparing the likelihood of ${ {\mathcal M} }_{{\rm{sym}}}$ with models involving partitions into *K* = 2 subsets. Agreement with the regions boundary is excellent. Our choice of *K* = 2 is justified as we would expect that models corresponding to partitions into two subsets to be the closest ones to the model ${ {\mathcal M} }_{{\rm{sym}}}$. An explicit expression for these bounds is derived in SI, Sec. 3, and Extended Data Figs 2 and 3 depict that they also bound considerably well the region in which ${ {\mathcal M} }_{{\rm{sym}}}$ is the likeliest for *β* = 2.

**Figure 2:**
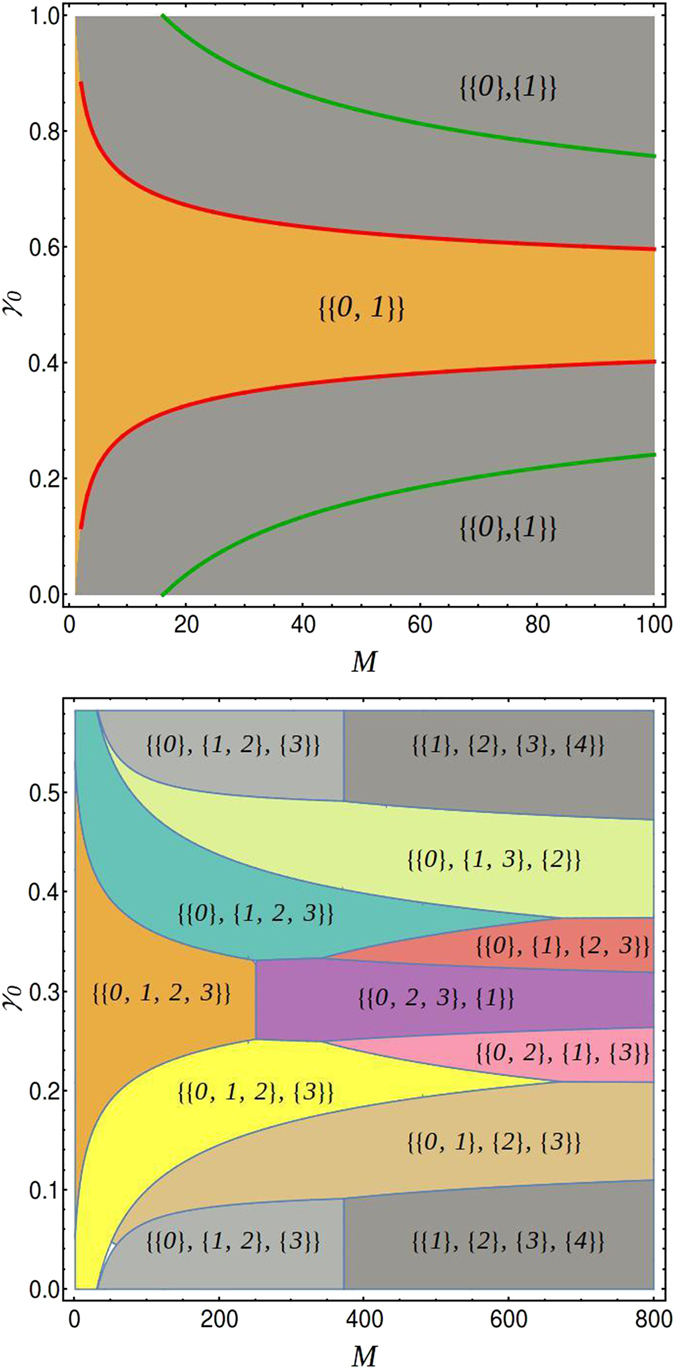
Comparison with NIST Suite test. Comparison of the bias allowed on a given sequence for it to be considered random using the NIST suite (upper panel) and our Bayesian method for randomness characterisation (lower panel).

For further benchmarking, we have compared our method against the NIST test suite^2^. The result is depicted in Fig. 2, as a function of the sequence length *M* and bias *b* employed to generate a 0. The upper panel on Fig. 2 shows the averaged number of tests passed when employing the NIST suite, while the lower one shows the frequency of ${ {\mathcal M} }_{{\rm{sym}}}$ being the likeliest, for *β* = 1, 2 and 3. We believe that our technique can contribute to test the quality of RNG in a more stringent form, since by applying a single test thrice (once for each value of *β*), we determined more precisely the random character of the sample of sequences.

As an application, we have tested our method in a bit sequence obtained experimentally from the differences in time detection in the process of spontaneous parametric down conversion (SPDC). Sequences generated via a SPDC photon-pair source have been shown to fulfil with ease the BN criterion, and to pass comfortably the NIST’s suite^1^. In the SPDC process a laser pump beam illuminates a crystal with a *χ* ^(2)^ nonlinearity, leading to the annihilation of pump photons and the emission of photon pairs, typically referred to as signal and idler^15^. Our experimental setup is shown in Extended Fig. 1 and we explain how to construct a 0 or 1 symbol from the detection signals in Section 1 of SI. We generated a 4 × 10^9^ bits sequence, so *β*_max_ ~ 4. When 1 ≤ *β* ≤ 3, we used all the possible models in the comparison, while, for computational ease, when *β* = 4, we restricted the model space to the 32, 768 models corresponding to *K* = 1 and *K* = 2 subsets (consider that ${B}_{{2}^{4}}={10}^{10}$). Our inference showed that ${ {\mathcal M} }_{{\rm{sym}}}$ was the likeliest model for every value of *β*.

As explained above, to achieve a full characterization of our QRNG as a random *source*, we need to go further from the model ranking based on the Bayes Factor and measure our certainty that ${ {\mathcal M} }_{{\rm{sym}}}$ is the true model governing the source. This (un)certainty quantification is the hallmark of Bayesian statistics, since $P({ {\mathcal M} }_{{\rm{sym}}}|\hat{s})$ represents the probability that modelling our QRNG as a random source is correct. Computing this posterior distribution directly from Bayes’ Theorem, Eq. 6, we arrive at the values shown in Table 1 for each *β*. The first three values are at least 0.95, but the corresponding to *β* = 4 is about 0.32, considerably smaller. However, this represents an improvement of order 10^4^ when compared with the initial value for the prior, ${P}_{0}({ {\mathcal M} }_{{\rm{sym}}})=1/32,768\approx 3.1\times {10}^{-5}$. Alternatively, we computed log_10_ BF_sym,*α*′_ for each value of *β*. The values reported in Table 1 correspond to the comparison of ${ {\mathcal M} }_{{\rm{sym}}}$ and the second likeliest model, hence the inequality for *β* > 2. These two criteria combined lead us to conclude that there is decisive evidence for our hypothesis that ${ {\mathcal M} }_{{\rm{sym}}}$ is the underlying model driving our source, thus verifying that the photonic RNG is strictly random in the sense described in the article.

**Table 1:** Posterior $P({ {\mathcal M} }_{{\rm{sym}}}|\hat{s})$ calculated for a dataset of 4 × 10^9^ bits.

*β*	${\boldsymbol{P}}({{\boldsymbol{ {\mathcal M} }}}_{{\bf{sym}}}|\hat{{\boldsymbol{s}}})$	log_10_ BF_sym,*α*′_
1	0.99993	4.15
2	0.99927	≥3.55
3	0.95374	≥1.84
4	0.31862	≥3.16

From a more general perspective, we propose that $P({ {\mathcal M} }_{{\alpha }^{(K)}}|\hat{s})$ quantifies our certainty on the hypothesis that a sequence $\hat{s}$ was generated using the biases on strings associated with *α* ^(*K*)^. Because Bayesian methods entails a model’s generalizability^9, 10^, the likeliest model provides a characterization of the source of $\hat{s}$. All partitions can be identified with standard computational packages, although it can be computationally demanding for sequences of ~10^10^ bits. In any case, once a partition is given, its model’s likelihood is easily found using Eq. (4). A simplified analysis can be performed with the BN-type bounds given in Section 3 of the SI, which also leads to more stringent criteria than other approaches.

## Electronic supplementary material

Supplementary information
