# Supplementary material for: Improving randomness characterization through Bayesian model selection
Source: Sci Rep. 2017 Jun 8;7:3096. doi: 10.1038/s41598-017-03185-y (PMC5465194; doi:10.1038/s41598-017-03185-y)
Supplement: Supplementary file 1 — Supplementary information [file 41598_2017_3185_MOESM1_ESM.pdf]

# Improving randomness characterization through Bayesian model selection

Rafael Díaz-H.R.[1], Aldo Solís[2], Alí M. Angulo Martínez[2], Alfred B. U'Ren[2], Jorge G. Hirsch[2], Matteo Marsili[3] & Isaac Pérez Castillo[1,4,*]

[1] *Instituto de Física, Universidad Nacional Autónoma de México. Apdo. Postal 20-364, Cd. Mx., Mexico, C.P. 04510*

[2] *Instituto de Ciencias Nucleares, Universidad Nacional Autónoma de México, Apdo. Postal 70-543, Cd. Mx., Mexico, C.P. 04510*

[3] *The Abdus Salam International Centre for Theoretical Physics, Strada Costiera 11, 34151 Trieste, Italy*

[4] *London Mathematical Laboratory, 14 Buckingham Street, London WC2N 6DF, United Kingdom*

## 1 Experimental Setup and conversion to a sequence of random bits.

The quantum state of the emitted photon pairs can be written as $|\Psi\rangle = |\text{vac}\rangle + \eta|\Psi_2\rangle$ in terms of the vacuum $|\text{vac}\rangle$, the two-photon component $|\Psi_2\rangle$, and of a constant $\eta$ related to the conversion efficiency. Under the assumptions a continuous-wave, plane-wave pump $|\Psi_2\rangle$ may be expressed as[1]

$$|\Psi_2\rangle = \int d\omega \int d\mathbf{k}^{\perp} F(\omega, \mathbf{k}^{\perp}) |\omega, \mathbf{k}^{\perp}\rangle_s |\omega_p - \omega, -\mathbf{k}^{\perp}\rangle_i, \tag{1}$$

written in terms of a joint amplitude function $F(\omega, \mathbf{k}^{\perp})$, and where $|\omega, \mathbf{k}^{\perp}\rangle_\mu$ represents a single-photon Fock state with frequency $\omega$ and transverse wavevector $\mathbf{k}^{\perp}$ for mode $\mu$, with $\mu = s, i$ for the

signal ($s$) and idler ($i$). In writing the two-photon state, we have assumed that the parametric down-conversion process is in the spontaneous regime, so that the appearance of multiple-pair events can be neglected. This assumption is valid if the parametric gain is sufficiently low; experimentally, we restrict the pump power so that the process remains spontaneous. In all likelihood, a similar experiment and analysis carried out in the high-gain, stimulated regime would yield different results from those presented on this paper.

The state in Eq. (1) is entangled since it cannot be factored into a direct product of separate states $|S\rangle$ (signal) and $|I\rangle$ (idler) as $|\Psi\rangle = |S\rangle|I\rangle$. While in many works based on SPDC photon pairs entanglement is the key resource, in our case we exploit instead the random times of emission (and detection) of signal and idler photons.

We have used a pump beam from a diode laser (DL407) centred at $407$nm with $\sim$ $60$mW power, and as nonlinear medium a $\beta$ barium borate (BBO) crystal of 1mm length; see Extended Data Figure 1. The BBO crystal, which is negative uniaxial, was cut so that the angle subtended by the optic axis with respect pump beam axis is $\theta_{\text{pm}} = 29.2°$ which yields phase matching for the generation of frequency-degenerate, non-collinear photon pairs. Signal and idler photons are emitted on diametrically opposed portions of an emission cone centred on the pump beam axis, with a $3.6°$ half opening angle. Pump photons are suppressed by transmitting the signal and idler modes through a long-pass filter which transmits wavelengths $\lambda > 488$nm (F1), followed by a bandpass filter centred at $800$nm with a $40$nm bandwidth (F2).

A halfwaveplate (HWP2) and a polarising beam splitter (PBS) are placed on the signal arm

so that the signal photon is transmitted or reflected with 50/50 probability. Each of the idler, reflected signal and transmitted signal collection modes is defined by an $f = 8$mm focal length aspheric lens (L1, L2 and L3) which focuses incoming light into the core of a multi-mode fibre with a $50\mu$m diameter (MMF1, MMF2 and MMF3). The plane defined by the collection fibres is chosen for convenience to be parallel to the optical table. By monitoring coincidences between the reflected signal and idler modes, on the one hand, and between the transmitted signal and idler modes, on the other hand, we are able to probabilistically exclude double (and multiple) pair events.

Each of the three photon-collection fibres leads to a silicon-based avalanche photodiode (APD1, APD2 and APD3), which emits an electronic TTL pulse for each detection event. The times of arrival of these pulses are monitored with a time to digital converter (TDC; id800 from IdQuantique), or time-tagger, with a resolution of 81 ps. The TDC produces three time series containing the time of arrival data for each of the idler ($i_n$), and transmitted ($s_n^t$) and reflected ($s_n^r$) signal channels. We generate by post-processing the two time series defined as $c_n^t = s_n^t \times i_n$, and $c_n^r = s_n^r \times i_n$, corresponding to those bins for which there are coincident detection events between the (reflected or transmitted) signal and idler channels. A sequence of bits is generated by comparing the differences in time detection with a fully regular time series *with the same number of events per second*. A value of $1$ is assigned if the time of detection is smaller than the corresponding time in the regular time series, and a value of $0$ otherwise[2].

We have checked on the efficiency of our QRNG in our experimental setup. According to our data, the efficiency based on the SPDC is 240 kilocounts per second in each channel. If only those events in which the signal and the idler photon are detected in coincidence are registered, the efficiency of random number generation is reduced to 27 kilocounts per second. Moreover, our experimental setup is such that we are able to discriminate four-photon versus two-photon events. This is achieved by noticing that, first of all, we have used a pump power such that the rate of four-photon generation is essentially negligible: less than $0.2\%$ according to our data. Secondly, in one of the SPDC arms we have placed a beamsplitter so that by discarding those events in which both APD's in that arm click, we can eliminate all the events in which events are detected in same time bin in the three detectors.

## 2 Derivation of Jeffreys Prior and Model's evidence

The idea of the Jeffreys prior is to take into account model indistinguishability from a point of view of a statistical sample. Based on Sanov's theorem[5] we know that the volume of models which are indistinguishable is inversely proportional to the square root of the determinant of the Fisher information matrix. This idea of measuring relevant volumes across models, but using a graining approach has also been explored previously[3,4] in a rigorous geometric treatment. Note that in this case, our parameters are the $\theta$'s of which only the (say) first $K-1$ are independent due to the normalization requirement. Then, considering a model $\mathcal{M}_{\alpha(K)}$ – also obviating the index $\ell$ in the partition, as we did in the main text – we have the following minus log-likelihood

$$-\log P\left(\hat{s}|\mathcal{M}_{\alpha(K)},\{\theta_r\}\right) = -\sum_{r=1}^{K} k_{\omega^{(r)}} \log\left(\frac{\theta_r}{|\omega^{(r)}|}\right) .$$

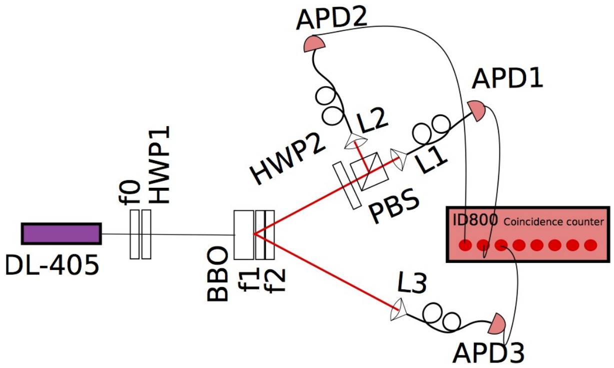


Extended Data Figure 1: **Experimental Setup**. A pump laser beam centred at 407nm (DL407) incides into nonlinear BBO crystal. The signal and idler generated photons are emitted at diametrically opposed portions of an emission cone which yields phase matching for frequency-degenerate non-collinear photon pairs. A polarising beam splitter (PBS) and a Half wavelength plate (HWP2) are placed at the signal portion of the cone so this photon can be transmitted or reflected with a 50/50 probability, the reflected and transmitted signal and idler photons are collected into multimode fibers that lead to avalanche photodiodes(APD1,2,3) which emit a TTL pulse for each detection event.

From here we derive the Fisher information matrix $J_{ab}$ for $a, b = 1, \ldots, K$, using the fact that $\frac{\beta}{M}\mathrm{E}[k_{\omega^{(r)}}] = \theta_r$,

$$J_{ab}(\theta) = -\frac{\beta}{M}\mathrm{E}\left[\frac{\partial^2}{\partial\theta_a\partial\theta_b}\log P\left(\hat{s}|\mathcal{M}_{\alpha^{(K)}}, \{\theta_r\}\right)\right] = \frac{1}{\theta_a}\delta_{a,b} + \frac{1}{\theta_K}\,,$$

where $\mathrm{E}[\cdots]$ denotes the expected value. Its determinant is simply $\det[J_{ab}(\theta)] = \frac{1}{\prod_{r=1}^{K}\theta_r}$. From here we have the following expression for Jeffreys prior:

$$P_{\mathrm{Jef}}(\theta) = \frac{\Gamma\left(\frac{3K}{2}\right)}{\Gamma^K\left(\frac{3}{2}\right)}\prod_{r=1}^{K}\theta_r^{1/2}\,, \tag{2}$$

where the normalization factor comes from:

$$\int\left[\prod_{r=1}^{K}d\theta_r\right]\left[\prod_{r=1}^{K}\theta_r^{1/2}\right]\delta\left(\sum_{r=1}^{K}\theta_r - 1\right) = \frac{\Gamma^K\left(\frac{3}{2}\right)}{\Gamma\left(\frac{3K}{2}\right)}\,.$$

Notice that in this case the Jeffreys prior always behaves as a proper one, that is, it is normalizable.

Finally, a similar integration shows that the model's evidence is given by[6]

$$\begin{aligned} P\left(\hat{s}|\mathcal{M}_{\alpha^{(K)}}\right) &= \int\left[\prod_{r=1}^{K}d\theta_r\right]P_{\mathrm{Jef}}(\theta)P\left(\hat{s}|\mathcal{M}_{\alpha^{(K)}}, \{\theta_r\}\right) \\ &= \frac{\Gamma\left(\frac{3K}{2}\right)}{\Gamma^K\left(\frac{3}{2}\right)}\prod_{r=1}^{K}\left(\frac{1}{|\omega^{(r)}|}\right)^{k_{\omega^{(r)}}}\frac{\prod_{r=1}^{K}\Gamma\left(\frac{3}{2} + k_{\omega^{(r)}}\right)}{\Gamma\left(\frac{3K}{2} + \frac{M}{\beta}\right)}\,. \end{aligned} \tag{3}$$

This allows us to identify the terms $\left(\frac{1}{|\omega^{(r)}|}\right)^{k_{\omega^{(r)}}}$ as the maximum likelihood estimators, and the ones involving the gamma functions as a measure of the relevant volume occupied in the parameter space, related to the model's complexity[3].

## 3 Borel-normality-type (BN-type) bounds

Suppose we are interested in discerning whether a given sequence is completely random or not. This means that we must look for the region in the parameter space $(\{\gamma_j\}_{j\in\Xi_\beta}, M)$ in which the

evidence of the symmetric model –corresponding to the partition of $\Xi_\beta$ into one subset– is bigger than the rest of the models. As the empirical frequencies $\{\gamma_j\}_{j\in\Xi_\beta}$ are grouped into $K$ subsets for a given partition $\alpha^{(K)}$, then the corresponding model has in effect $K-1$ free parameters $\{\gamma_{\omega^{(r)}}\}_{r=2}^{K}$. Recalling that we used the Bayes Factor as a decision rule in the main text, we can explore the conditions such that $\mathcal{M}_{\rm sym}$ is the likeliest by the behaviour of the log-likelihood ratio, $\log\left(\frac{P(\hat{s}|\mathcal{M}_{\rm sym})}{P(\hat{s}|\mathcal{M}_{\alpha^{(K)}})}\right)$.

To obtain a BN-type bound, we do the following: i) look for the values $\{\gamma^\star_{\omega^{(r)}}\}_{r=2}^{K}$ which extremize the log-likelihood ratio; ii) do an expansion around those values up to second order. We eventually obtain:

$$\begin{aligned}
&\log\left(\frac{\prod_{r=1}^{K}\left(|\omega^{(r)}|\right)^{\frac{M}{\beta}\gamma^\star_{\omega^{(r)}}}\Gamma^K\left(\frac{3}{2}\right)\Gamma\left(\frac{3K}{2}+\frac{M}{\beta}\right)}{2^M\Gamma\left(\frac{3K}{2}\right)\prod_{r=1}^{K}\Gamma\left(\frac{3}{2}+\frac{M}{\beta}\gamma^\star_{\omega^{(r)}}\right)}\right)\\
&=\frac{1}{2}\left(\frac{M}{\beta}\right)^2\sum_{r,r'=2}^{K}\left(\gamma_{\omega^{(r)}}-\gamma^\star_{\omega^{(r)}}\right)\left(\gamma_{\omega^{(r')}}-\gamma^\star_{\omega^{(r')}}\right)\\
&\times\left[\delta_{r,r'}\psi_1\left(\frac{3}{2}+\frac{M}{\beta}\gamma^\star_{\omega^{(r)}}\right)+\psi_1\left(\frac{3}{2}+\frac{M}{\beta}\left(1-\sum_{r=2}^{K}\gamma^\star_{\omega^{(r)}}\right)\right)\right], \qquad (4)
\end{aligned}$$

where the $\gamma^\star$-unknowns obey the following set of equations

$$\psi\left(\frac{3}{2}+\frac{M}{\beta}\gamma^\star_{\omega^{(r)}}\right)-\psi\left(\frac{3}{2}+\frac{M}{\beta}\left(1-\sum_{r=2}^{K}\gamma^\star_{\omega^{(r)}}\right)\right)=\log\left|\frac{\omega^{(r)}}{\omega^{(1)}}\right|, \qquad r=2,\ldots,K. \qquad (5)$$

Here the function $\psi_n(x)$ is the polygamma function of order $n$, with $\psi(x)\equiv\psi_0(x)$. As the symmetric model is the one that corresponds to no-free parameters, one could reasonable assume that the models which are closer to $\mathcal{M}_{\rm sym}$ are those which correspond a single free parameter. This, in turn, corresponds to subfamilies of partitions into two subsets of lengths $\{2^\beta-q,q\}$ for $q=1,\ldots,2^\beta/2$, which will have aggregate frequencies $1-\gamma_{|q|}$ and $\gamma_{|q|}$ respectively. This is also

justified by the lower panel of Figure 1 in the main text, which shows that the transition from $K = 1$ to a bigger value should necessarily go through a region where a model with $K = 2$ is likelier than $\mathcal{M}_{\rm sym}$. Applying this to the set of Eqs. (4) and (5) we obtained that $\left|\gamma_q - \gamma^\star_{|q|}\right| \leq \frac{\sqrt{2}\beta}{M}\mathcal{W}(\gamma^\star_{|q|})$ with the function $\mathcal{W}(\gamma^\star_{|q|})$ defined as

$$\mathcal{W}(\gamma^\star_{|q|}) \equiv \sqrt{\frac{\log\left(\frac{\Gamma^2(3/2)\Gamma(3+M/\beta)\left(2^\beta-q\right)^{\frac{M}{\beta}(1-\gamma^\star_{|q|})}q^{\frac{M}{\beta}\gamma^\star_{|q|}}}{2^M\Gamma(3)\Gamma\left(1+\frac{M}{\beta}\gamma^\star_{|q|}\right)\Gamma\left(1+\frac{M}{\beta}(1-\gamma^\star_{|q|})\right)}\right)}{\psi_1\left(\frac{3}{2}+\frac{M}{\beta}\gamma^\star_{|q|}\right)+\psi_1\left(\frac{3}{2}+\frac{M}{\beta}\left(1-\gamma^\star_{|q|}\right)\right)}}\,, \tag{6}$$

where $\gamma^\star_{|q|}$ are the aggregated frequencies of a subset of size $q$ satisfying the extremisation condition

$$\psi\left(\frac{3}{2}+\frac{M}{\beta}\gamma^\star_{|q|}\right)-\psi\left(\frac{3}{2}+\frac{M}{\beta}\left(1-\gamma^\star_{|q|}\right)\right)=\log\left(\frac{q}{2^\beta-q}\right)\,, \tag{7}$$

for $q = 1, \ldots, 2^\beta/2$.

In particular, for $\beta = 1$, there is only one model to compare to $\mathcal{M}_{\rm sym}$, which precisely corresponds to $K = 2$. Here, the solution of (7) is exactly $\gamma^\star_{|1|} = 1/2$, which provides the following bound:

$$\left|\gamma_1 - \frac{1}{2}\right| \leq \frac{1}{M}\sqrt{\frac{\log\left(\frac{2^{-M}\Gamma(3+M)}{\Gamma^2\left(\frac{3}{2}+\frac{M}{2}\right)}\right)}{\psi_1\left(\frac{3}{2}+\frac{M}{2}\right)}}\,, \tag{8}$$

This is the formula we used to draw the red curves in the top panel of Figure 1 of the main text together with the exact diagram. Agreement for this simple bound is excellent compared to the exact formulas, and rather different as compared to the one of BN. For the case $\beta = 2$, one must solve the set of equations numerically to evaluate the bounds. They work reasonably well in the parameter space and much better than the BN bounds as shown in Extended Data Figure 2. Notice that in these figures we only depict two regions in the parameter space: the orange one corresponds to the region in which the symmetric model is likeliest, while the grey-filled area in which it is not.

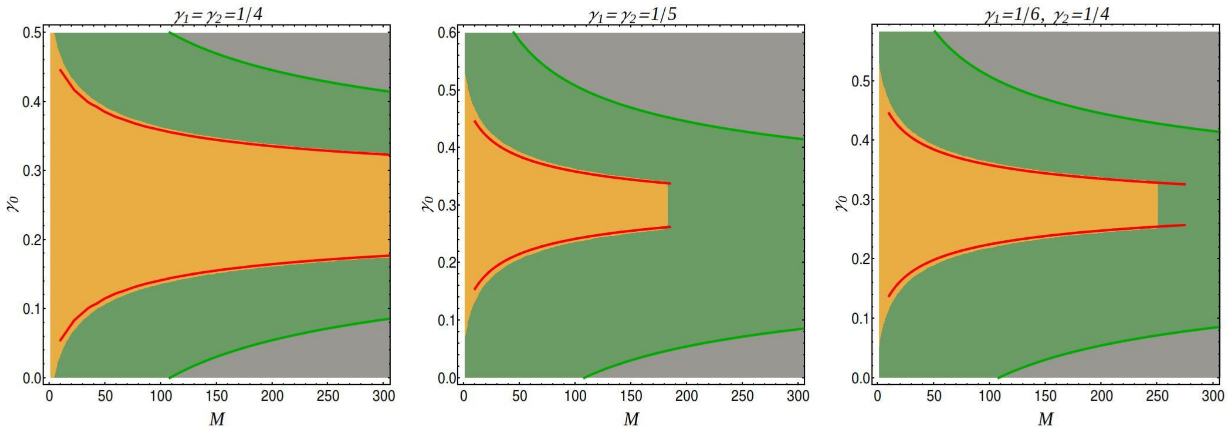


Extended Data Figure 2: **BN-type bounds**. Phase diagram of model selection for the 15 models for $\beta = 2$ and various fixed values of $\gamma_1$ and $\gamma_2$. Here the orange filled area represents the region in which model $\mathcal{M}_{\text{sym}}$ is the likeliest, while the grey filled area represents the region in the parameter space in which any other model is the likeliest. Solid red lines represent the BN-type bounds. We also compare with the BN bounds (green filled region). Notice that for the second and the third case, the BN bounds also provides a bound for $M$ given by the solution of $|1/4 - 1/5| = \sqrt{\frac{\log_2(M)}{M}}$ and $|1/4 - 1/6| = \sqrt{\frac{\log_2(M)}{M}}$, respectively.

These previous bounds have the disadvantage of needing to solve the system (7) numerically. However, looking at the set of Eqs. (5) we notice that there is a particular set of partitions for which its solution is particularly simple, namely when the system is solved using only equi-partitions, that is, partitions into subsets of the same size. With this restriction, it is possible to find simpler, less restrictive bounds, yet tighter than the ones derived from other methods. Suppose that we look at partitions into $K$ subsets. Within this family (and of course for even $K$) we will have a subfamily of equi-partitions. For them we have that $|\omega^{(r)}| = |\omega^{(1)}| = \frac{2^\beta}{K}$ and therefore $k^\star_{\omega^{(r)}} = M/(\beta K)$ and $\gamma^\star_{\omega^{(r)}} = 1/K$. In particular, for the model corresponding to a partition into $K = 2^\beta$ subsets, the formula (4) becomes:

$$\sum_{i\leq j=1}^{2^\beta-1}\left(\gamma_i - \frac{1}{2^\beta}\right)\left(\gamma_j - \frac{1}{2^\beta}\right) = \left(\frac{\beta^2 \log\left(\frac{2^{-M}\Gamma\left(\frac{3}{2^{1-\beta}}+\frac{M}{\beta}\right)}{\Gamma\left(\frac{3}{2^{1-\beta}}\right)\Gamma^{2^\beta}\left(\frac{3}{2}+\frac{M}{\beta 2^\beta}\right)}\right)}{M^2\psi_1\left(\frac{3}{2}+\frac{M}{\beta}\right)}\right), \tag{9}$$

a bound which, unlike the one of Borel-normality, couples all the empirical frequencies. Results of these broader bounds are plotted in Extended Data Figure 3.

## 4 Some examples for the evidence

In this section, we illustrate, with some specific examples, the formulae Eq. (4) for the particular case of $\beta = 2$. Because explicit reference to specific partitions is made, we will use the full notation $\alpha_\ell^{(K)}$, although there is no natural order to assign the index $\ell$. In this case we have the following partitions of $\Xi_{\beta=2}$, corresponding to 15 models: a partition into $K = 1$ subset (symmetric model) which corresponds to $\alpha_1^{(1)} = \{\{0,1,2,3\}\}$. There are $\left\{ {4 \atop 2} \right\} = 7$ ($\left\{ {a \atop b} \right\}$ denotes the Stirling number of second kind) partitions with $K = 2$ subsets, which are: $\alpha_1^{(2)} = \{\{0\},\{1,2,3\}\}$,

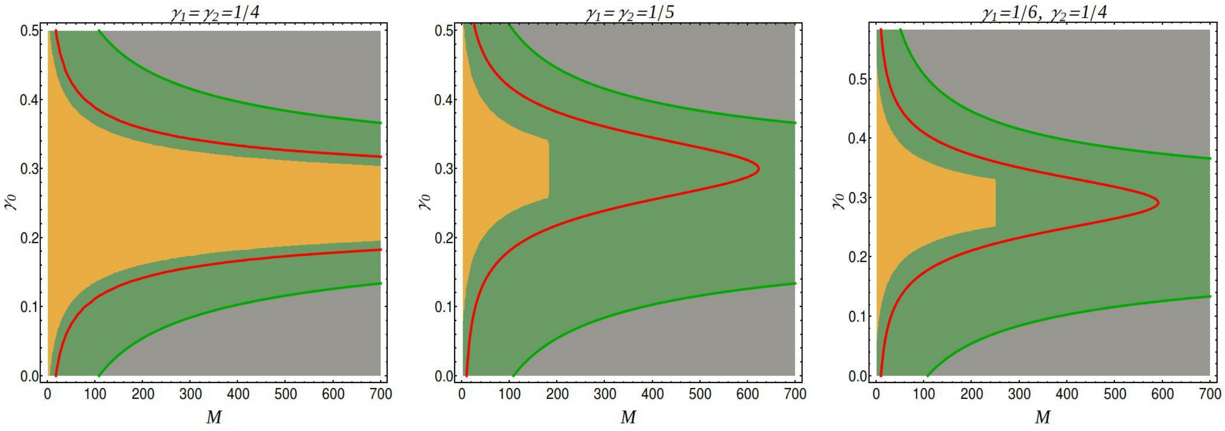


Extended Data Figure 3: **BN-type bounds**. Phase-type diagram for model selection for $\beta = 2$ and comparison between the bounds given by the simple formula (6) (solid red line) and the Borel-normality bounds (solid green line).

$\alpha_2^{(2)} = \{\{0,1\},\{2,3\}\}$, $\alpha_3^{(2)} = \{\{0,2,3\},\{1\}\}$, $\alpha_4^{(2)} = \{\{0,1,2\},\{3\}\}$, $\alpha_5^{(2)} = \{\{0,3\},\{1,2\}\}$, $\alpha_6^{(2)} = \{\{0,1,3\},\{2\}\}$, $\alpha_7^{(2)} = \{\{0,2\},\{1,3\}\}$. We have $\left\{ {4 \atop 3} \right\} = 6$ partitions into $K = 3$ subsets: $\alpha_1^{(3)} = \{\{0\},\{1\},\{2,3\}\}$, $\alpha_2^{(3)} = \{\{0\},\{1,2\},\{3\}\}$, $\alpha_3^{(3)} = \{\{0\},\{1,3\},\{2\}\}$, $\alpha_4^{(3)} = \{\{0,1\},\{2\},\{3\}\}$, $\alpha_5^{(3)} = \{\{0,2\},\{1\},\{3\}\}$, $\alpha_6^{(3)} = \{\{0,3\},\{1\},\{2\}\}$. And, finally, one partition $\alpha_1^{(4)} = \{\{0\},\{1\},\{2\},\{3\}\}$ into $K = 4$ subsets.

An example of the evidence, of the model associated to partition e.g. $\alpha_1^{(3)}$ is

$$P\left(\hat{s}|\mathcal{M}_{\alpha_1^{(3)}}\right) = \frac{\Gamma\left(\frac{9}{2}\right)}{\Gamma^3\left(\frac{3}{2}\right)} \left(\frac{1}{2}\right)^{k_{\omega^{(3)}}} \frac{\Gamma\left(\frac{3}{2}+k_{\omega^{(1)}}\right)\Gamma\left(\frac{3}{2}+k_{\omega^{(2)}}\right)\Gamma\left(\frac{3}{2}+k_{\omega^{(3)}}\right)}{\Gamma\left(\frac{9}{2}+\frac{M}{2}\right)} \tag{10}$$

$$= \frac{\Gamma\left(\frac{9}{2}\right)}{\Gamma^3\left(\frac{3}{2}\right)} \left(\frac{1}{2}\right)^{k_2+k_3} \frac{\Gamma\left(\frac{3}{2}+k_0\right)\Gamma\left(\frac{3}{2}+k_1\right)\Gamma\left(\frac{3}{2}+k_2+k_3\right)}{\Gamma\left(\frac{9}{2}+\frac{M}{2}\right)}, \tag{11}$$

where $k_{\omega^{(1)}}$ ($k_{\omega^{(2)}}$) is the number of occurrences of string $\{0\} = \{00\}$ (resp. $\{1\} = \{01\}$), and $k_{\omega^{(3)}}$

is the added number of occurrences of the strings $\{2\} = \{10\}$ and $\{3\} = \{11\}$ in the sequence of bits. An equivalent expression with the individual frequencies $k_j$ of the $j$-th string is also given for clarity.

## 5 On the choice for the Prior of models

Since in this work our particular goal is to assess the randomness of a given sequence with a general applicable method, it would be convenient to obtain a criterion as sharp as possible when no previous knowledge of the source producing the data is given. Morevover, another desirable property would be that no particular type of sequence is preferred over the rest, or in other words, we would like to reproduce a distribution on datasets that resembles closely a uniform prior distribution over them. As we will justify here, those two features can be achieved by choosing a uniform prior distribution on the models, that is, for a fixed $\beta$, $P_0(\mathcal{M}_\alpha) = \frac{1}{B_{2^\beta}}$, with $B_n$ the $n$-th Bell number. Indeed, this results in a distribution on sequences for which the unbiased ones are the most unlikely.

Indeed, first of all, we need to relate the prior distribution on models $P_0(\mathcal{M}_\alpha)$ with the prior distribution on sequences $P_0(\hat{s})$. This can be done by computing the marginal of their joint distribution, $P_0(\hat{s}) = \sum_\alpha P(\hat{s}|\mathcal{M}_\alpha)P_0(\mathcal{M}_\alpha)$. We want to show that a uniform prior on models results into an expression of $P_0(\hat{s})$ that penalizes unbiased sequences. To be specific, let us analyse the case of $\beta = 1$, for which there are only two possible models, and hence $P_0(\mathcal{M}_\alpha) = \frac{1}{2}$. Using

Eqs. (4) and (5) from the main text to calculate the above marginal, we obtain the following

$$P_0(\hat{s}) = \frac{1}{2}\left[\frac{1}{2^M} + \frac{\Gamma(3)\Gamma\left(k_0+3/2\right)\Gamma\left(k_1+3/2\right)}{\Gamma\left(M+3\right)\Gamma^2(3/2)}\right]. \tag{12}$$

From this expression, we can see that under the assumption of uniform prior distributions over *models*, we obtain two terms for the prior distribution on *datasets*: the first one is independent on the frequency of strings, while the second term adds a non-negative contribution that depends explicitly on such frequencies. However, this second term is just the $B$ function, whose global minimum is achieved when $k_0 = k_1 = M/2$. Thus unbiased sequences for which presumably $k_0 \approx k_1$ are unfavored with this assumption.

An analogous argument follows straightforwarldy for larger values of $\beta$. It is also worth mentioning that were we to assume directly that $P_0(\hat{s}) = \frac{1}{2^M}$, the only compatible prior over models would be $P_0(\mathcal{M}_\alpha) = \delta_{\mathrm{sym},\alpha}$.


1. Vicent, L. E. *et al.* Design of bright, fiber-coupled and fully factorable photon pair sources. *New Journal of Physics* **12**, 093027 (2010).

2. Solis, A. *et al.* How random are random numbers generated using photons? *Physica Scripta* **90**, 074034 (2015).

3. Myung, I. J., Balasubramanian, V. & Pitt, M. A. Counting probability distributions: Differential geometry and model selection. *Proceedings of the National Academy of Sciences* **97**, 11170–11175 (2000).

4. Balasubramanian, V. Statistical inference, occam's razor, and statistical mechanics on the space of probability distributions. *Neural computation* **9**, 349–368 (1997).

5. Mastromatteo, I. On the typical properties of inverse problems in statistical mechanics. *PhD Thesis* (2013).

6. MacKay, D. J. Bayesian interpolation. *Neural computation* **4**, 415–447 (1992).
